# In vivo determination of domestic quail (*Coturnix japonica*) muscle development by ultrasonography as a function of energy levels

**DOI:** 10.1038/s41598-023-29233-4

**Published:** 2023-02-13

**Authors:** Thailine Santana Santos, Isis Nayara Santana Alves, Jucélia dos Santos Araujo, Ananda Santos de Assis, Valdir Ribeiro Júnior, Claudson Oliveira Brito, Camilla Mendonça Silva, Anselmo Domingos Ferreira Santos, Leandro Teixeira Barbosa, Vittor Tuzzi Zancanela, Gregório Murilo de Oliveira Júnior

**Affiliations:** 1 Depto de Zootecnia, Universidade Federal de Sergipe, Av. Marechal Rondon S/N, Rosa Elze, 49100-000 São Cristóvão, SE Brasil; 2 Depto de Zootecnia, Universidade Federal de Sergipe, Campus Sertão, 49680-000 Nossa Senhora da Glória, SE Brasil; 3 Depto de Medicina Veterinária, Universidade Federal de Sergipe, Av. Marechal Rondon S/N, Rosa Elze, 49100-000 São Cristóvão, SE Brasil

**Keywords:** Animal physiology, Software

## Abstract

The aim was to evaluate the growth and meat production and some meat quality characteristics of domestic quail (*Coturnix japonica*) as a function of metabolizable energy (ME) levels in the diet and to adjust predicting equations in ratio to area *pectoralis major* muscle of the carcass through the ultrasound. Two hundred and seventy mixed sex quail from 7 to 49 days old were distributed in three treatments (7 to 21 days old, diets with 2700; 2900 and 3100 kcal ME/kg; from 21 to 49 days, diets with 2900, 3050 and 3200 kcal of ME/kg), with five replications per treatment. Ultrasonography was performed at 21; 35 and 49 days of age in the *pectoralis major* muscle to determine prediction equations through multiple linear regression. Feed intake (FI) and feed efficiency (FE) were linearly influenced (*P* < 0.05) by energy levels in both phases evaluated. Regarding sensory analysis, there was no interference of ME levels on sensory attributes. The equation for predicting breast area was Y = 0.00271*ME + 0.25411*Age-9.58002, R^2^ = 74.25%. It is concluded that increasing the energy level of the feed from 2700 to 3100 kcal ME/kg reduces FI and improves the FE of quail. The increase in carcass fat from 35 days of age does not harm the physical and sensory characteristics of the meat. The ultrasonography in vivo of the *M. pectoralis major*, considering the age and energy level of the diet, made it possible to predict the pectoral muscle are a with higher reliability.

## Introduction

Breeding quails for meat is a promising activity^1^; factors such as precocity in production, sexual maturity, high productivity and low initial investment, followed by a rapid financial return, are decisive factors for the growth of the activity.

According to El-Bahr et al.^2^, the meat quails represent an alternative food; however, in some countries is the main source of animal protein in the diet. For quail production to continue expanding, it is necessary to provide a quick financial return to the producer and maintain affordable products and quality for consumers. Factors that directly depend of the correct balance of the diets provided to these birds.

In this context, the energy concentration in quail diets affects the growth rate and the deposition of protein and fat in the carcass^3^. Therefore, the levels of metabolizable energy must be adequate in the diet formulations, as their levels can directly affects the production, meat quality and production cost. According to Muniz et al.^4^, the supply of metabolizable energy is essential for the maintenance, growth, and reproduction of animals. Thus, the levels used in the formulations can directly affect the composition of the carcasses, especially the fat content.

Santos et al.^3^ inform that diets with 3050 kcal of ME/kg increment the abdominal fat deposition of the carcasses of domestic meat quails. While Lima et al.^5^ did not observe an increase in abdominal fat in domestic meat quail fed 2950 kcal of ME/kg.

Thus, the ultrasound technique can be used to measure the effect of diets on the *pectoralis major* muscle development and on the fat deposition in the carcasses of domestic quails. Ultrasonography used in vivo is already a technology widely used in research aimed at large and medium-sized farm animals, allowing the observation of body development and measuring, mainly, the development of muscle and adipose tissue in vivo^6^. However, studies on the use of the ultrasound device in birds are still incipient and scarce.

Using this technology in quail can bring many benefits to the production chain, for example, in the selection and in the slaughtering time prediction. According to Teixeira et al.^7^, the ultrasound technique allows real-time assessment of body development and carcass composition with precise and accurate measurements.

Based on this information, the hypothesis adopted in the study was that the increase in the energy density of the diets could affect the performance and carcass characteristics of domestic quail and to predict through in vivo ultrasound muscle development as a function of metabolizable energy levels.

Therefore, the aim was to evaluate the growth, performance, and some meat quality characteristics of dual-purpose domestic quail (*Coturnix japonica*) as a function of metabolizable energy (ME) levels in the diet; and to determine the area of the M. *pectoralis major* of the carcasses through of ultrasound and prediction equations.

## Results

### Performance

During the experiment, no differences (*P* > 0.05) of treatments were observed on the BW or the AWG of quails at 21, 35 days or at 49 days of age (Table 1). However, FI was reduced (*P* < 0.01) and FE improved (*P* < 0.01) with increasing levels of ME from 2700 to 3200 kcal in the period from 7 to 21 days, as well as in the period from 7 to 35 and from 7 to 49 days.

**Table 1:** Performance of European quail meat as a function of metabolizable energy levels (kcal ME/kg).

Parameters [g]	ME levels (kcal/kg)			SE	P value
	2700	2900	3100		
7–21 days age					
BW initial [g]	25.14	25.10	25.08	0.17	–
BW [g]	147.67	150.09	149.96	1.43	0.45
AWG [g/period]	122.47	125.00	124.77	1.49	0.44
FI [g/period]	181.07^b^	174.48^b^	145.83^a^	3.54	< 0.01
FE	0.68^b^	0.72^b^	0.87^a^	0.02	< 0.01

ME Levels (kcal/kg)					
	2900	3050	3200		
7–35 days age					
BW initial [g]	229.79	226.67	226.49	1.80	0.38
BW [g]	201.47	201.39	204.60	1.85	0.40
AWG [g/period]	434.29^b^	426.95^b^	390.67^a^	6.00	< 0.01
FI [g/period]	0.46^b^	0.42^b^	0.52^a^	0.01	< 0.01
7–49 days age					
BW initial [g]	291.43	286.44	284.09	5.08	0.59
BW [g]	258.95	257.89	266.42	5.62	0.54
AWG [g/period]	826.12^b^	779.60^ab^	749.48^a^	12.34	< 0.01
FI [g/period]	0.31^b^	0.33^b^	0.36^a^	0.01	< 0.01

SE: Standard error of the mean; Means followed by different letters on the same line differ by Tukey's test at 5% probability.

*BW* body weight, *AWG* average weight gain, *FI* feed intake, *FE* Feed efficiency.

### Ultrasonography

It was possible to estimate prediction equations for the information by the ultrasonography method for the measurements of area, width and depth in the longitudinal and transversal reading of the M. *pectoralis major* (Table 2). It can be seen that the transversal-sectional reading area had a higher coefficient of determination (R^2^ = 74.25), demonstrating greater representativeness to the muscle measurement. For muscle depth were found R^2^ = 46.41. However, it was not possible to delimit the area of the quail adipose tissue in any of the proposed portions, and therefore, it was not considered in the discussion.

**Table 2:** Prediction equations to determine the area, width and depth of the M. *pectoralis major* as a function of quail age and metabolizable energy levels in the diets.

Variable	Equation	R^2^ (%)
Transversal reading measurements		
Area (cm^2^)	Y = 0.00271*ME + 0.25411*Age − 9.58002	74.25
Width (cm)	Y = 0.00015556*ME + 0.02587*Age + 5.91880	29.51
Depth (cm)	Y = 0.00006031*ME + 0.01265*Age + 0.59616	46.41
Longitudinal reading measurements		
Area (cm^2^)	Y = − 0.00010344*ME + 0.08873*Age + 1.80818	19.40
Width (cm)	Y = 0.00013368*ME-0.01170*Age + 5.19448	11.62
Depth (cm)	Y = 0.00007363*ME + 0.01255*Age + 0.55718	48.61

### Relative weight of organs

At 21 days of age, the relative weight of the liver was reduced (*P* < 0.01) at levels above 2900 kcal ME/kg ratio, while it increased the weight of the gizzard with increasing levels of metabolizable energy (*P* < 0.05). However, the other parameters were not influenced (Table 3).

**Table 3:** Relative weights of organs, intestine and fat abdominal as a function of metabolizable energy levels (ME) in the diet and production phase of European quail meat.

Weight [%]	ME (kcal/kg)			SE	P-Value
	2700	2900	3100		
21 days old					
Heart	0.75	0.81	0.75	0.03	0.37
Liver	4.30^a^	3.28^b^	3.43^b^	0.15	< 0.01
Pancreas	0.44	0.40	0.41	0.02	0.30
Proventriculus	0.69	0.71	0.77	0.03	0.25
Gizzard	2.96^b^	3.40^ab^	3.42^a^	0.11	0.03
Intestine	5.93	5.84	5.19	0.43	0.44
Abdominal fat	0.00	0.07	0.00	0.04	0.41

ME (kcal/kg)					
	2900	3050	3200		
35 days old					
Heart	0.86	0.90	0.84	0.03	0.47
Liver	2.40	2.14	2.12	0.18	0.51
Pancreas	0.29^a^	0.26^ab^	0.20^b^	0.02	< 0.01
Proventriculus	0.42^a^	0.40^ab^	0.33^b^	0.02	0.02
Gizzard	2.68^a^	2.51^ab^	2.23^b^	0.11	0.05
Intestine	3.23	3.28	3.37	0.20	0.89
Abdominal fat	0.35^a^	0.43^b^	1.26^b^	0.16	< 0.01
49 days old					
Heart	0.77	0.80	0.80	0.04	0.74
Liver	2.86	2.62	2.57	0.19	0.54
Pancreas	0.21	0.23	0.24	0.01	0.37
Proventriculus	0.40	0.36	0.37	0.04	0.68
Gizzard	1.92	2.35	2.11	0.12	0.08
Intestine	3.76	3.66	3.60	0.34	0.94
Abdominal fat	1.18^a^	1.69^b^	1.29^ab^	0.19	0.03

SE: Standard error of the mean; Means followed by distinct letters in the lines differ from each other by Tukey's test at 5% probability.

At 35 days, it was observed that the weight of the pancreas, proventriculus and gizzard were reduced (*P* < 0.05) when the inclusion of ME in the diets was increased, while the fat content was higher (*P* < 0.01) in the quail that consumed 3050 and 3200 kcal of ME/kg. At 49 days, the fat content was higher in the quail that consumed 3050 and 3200 kcal of ME/kg ratio.

### Body chemical composition

There were no differences between treatments (*P* > 0.05) for dry matter, crude protein and ether extract content at 21 days of age (Table 4). However, the mineral matter content was lower in quail that consumed diets above 2900 kcal ME/kg.

**Table 4:** Quail meat chemical composition as a function of different levels of metabolizable energy (ME).

Parameters [%]*	ME (kcal/kg)			SE	P valor
	2700	2900	3100		
21 days old					
DM	31.11	31.36	31.25	0.63	0.97
ASH	9.62^a^	8.92^b^	8.87^b^	0.18	0.02
CP	64.50	64.99	63.92	1.23	0.84
EE	19.81	18.17	19.03	1.08	0.60

ME (kcal/kg)					
	2900	3050	3200		
35 days old					
DM	35.47	35.76	35.77	0.31	0.77
ASH	9.14	9.22	8.66	0.29	0.36
CP	63.84^a^	58.56^b^	53.22^c^	0.97	< 0.01
EE	24.83	25.60	25.60	1.32	0.88
49 days old					
DM	39.32	40.31	39.92	0.79	0.70
ASH	7.17	6.96	6.85	0.22	0.58
CP	47.97	50.73	48.65	1.17	0.17
EE	35.08	35.40	33.68	0.94	0.46

SE: Standard error of the mean; Means followed by different letters differ from each other by Tukey's test at 5% probability.

*DM* dry matter, *CP* crude protein, *EE* ether extract.

*Values on based on natural matter.

At 35 days, it was observed that the increase from 2900 to 3200 kcal ME/kg in the diet negatively influenced the carcass protein content (*P* < 0.01), while it did not influence the other parameters. At 49 days of age, the quail maintained (*P* > 0.05) proportions of protein, fat and mineral matter, similar.

### Slaughter characteristics and meat quality

The carcass weights, and the initial (slaughter) and final 24 h *post mortem* pH, were not influenced (*P* > 0.05) by the energy levels of the ration. The increase in energy levels did not influence the carcass, breast, thigh + drumstick (leg) yield, but the wing yield was higher in the quails that consumed 3200 kcal of ME/kg (Table 5).

**Table 5:** Quail meat performance (49 days of age) and breast pH as a function of metabolizable energy levels (ME).

Parameter	ME (kcal/kg)			SE	P value
	2900	3050	3200		
Carcass weight [g]	179.52	186.60	179.18	6.11	0.63
Carcass yield [%]	65.80	67.53	67.47	1.03	0.42
Breast [%]	40.53	39.72	39.92	0.60	0.62
Thigh + drumstick [%]	23.03	23.60	23.51	0.37	0.51
Wing [%]	7.74^b^	7.63^b^	8.29^a^	0.18	0.03
pH [*p.m*]	5.85	5.81	5.76	0.04	0.28
pH [24 h *p.m*]	5.85	5.89	5.85	0.03	0.73

SE: Standard error of the mean; Means followed by different letters differ from each other by Tukey's test at 5% probability.

The description of the profile of the tasters who took part in the study showed that the majority were male (52%), aged between 18 and 28 years, and on the frequency of consumption of quail meat, it was observed that 45% of the tasters consume it annually or have never consumed, while 10% make the monthly consumption of quail meat. The results got showed that ME levels did not interfere with any of the sensory attributes evaluated, which consequently did not interfere (*P* > 0.05) with the acceptability of quail meat by the tasters (Table 6).

**Table 6:** Analysis of the sensory attributes of the breast fillet in relation to the levels of metabolizable energy (ME) in quail diet (49 days of age).

Attributes*	ME (kcal/kg)			P value
	2900	3050	3200	
Appearance	5.44 ± 0.17	5.78 ± 0.15	5.79 ± 0.15	0.21
Odor	5.85 ± 0.15	6.12 ± 0.12	5.89 ± 0.15	0.36
Flavor	6.02 ± 0.18	5.93 ± 0.17	6.02 ± 0.17	0.90
Softness	6.52 ± 0.16	6.61 ± 0.14	6.51 ± 0.18	0.87
Global Acceptance	5.95 ± 0.18	6.07 ± 0.17	6.00 ± 0.18	0.89

*Score for each attribute—Hedonic scale form where 1 represents: I disliked a lot and 8 represents: I liked a lot (Dutcosky^33^).

## Discussion

The increase in energy density in the diets of quails from 7 to 49 days of age did not provide significant effects on the BW and AWG of the birds. The level of 2700 kcal ME/kg ratio in the initial phase suffices to maintain the performance of quails since, in the initial phase, birds have a greater need for protein and amino acids because of rapid muscle growth^8^.

In addition, the maximum growth rate in quail usually occurs until around 27 days of life, when the energy demand rises. This was evidenced by Silva et al*.*^9^, who through the Gompertz equation they verified the inflection point of the quail growth curve close to 23 and 24 days of age for males and females, respectively, which reiterates the fact of maximum growth at 27 days.

The increase in the energy level of the diet promoted a reduction in the AFI of the birds; however, there was an improvement in FE, even though the BW changed neither in the period up to 35 or 49 days of age. According to Hu et al.^10^, energy acts as a regulator in feed intake, changing the speed of passage of the diet and intestinal motility in the digestive tract of animals. This can be confirmed, possibly, also by the greater production of cholecystokinin in birds that consume higher energy levels, as this hormone is stimulated by the presence and amount of lipids in the body, influencing the passage rate and use of food in the intestine^11^. As a result, the food remains longer in the digestive tract of birds and; therefore, the digestibility of the diet may be affected^12^.

These results corroborate those found by Muniz et al.^13^, evaluating ME levels different for growing quail, which also observed a significant effect on the reduction of feed intake with the increase of ME in the diet. This confirms the theory that birds regulate feed intake according to ME content in the diet.

Through the ultrasound technique the area M. *pectoralis major* in the transversal-sectional reading it was more effective in predicting the muscle area of quail, as they had higher reliability. This major factor that differentiates it from other measures, since the other equations were less accurate.

Because of what has been reported, the equation for the area of the *M. pectoralis major* in the transversal reading got a high coefficient of determination, possibly because it better expresses the assessed muscle and presents a greater representation of the measures analyzed. Muscle area is a more comprehensive measure, which indirectly measures muscle depth and width, factors that reflect the reliability of the equation.

Akbarnejad et al.^14^ worked with a genetic evaluation of carcass traits in Japanese quails using ultrasound and morphological measurements and observed that the estimation of the breast ultrasound image had a high genetic correlation with the estimation of morphological measurements performed after slaughter. The authors further stated that breast muscle ultrasound measurements could effectively predict breast weight and performance in Japanese quails. According to Simões et al.^15^, the use of reading in ultrasound devices to determine the volume of breast muscles can significantly reduce the costs of in vivo studies without decreasing the robustness in predicting carcass and breast yields, when compared to other techniques.

Finally, from the prediction equation and the level of metabolizable energy in the diet, the quail meat producer can identify when the birds will reach the ideal muscle development and thus define the slaughter of the birds in a more efficient way.

The low-fat content in the quail carcasses, as well as difficulties in reading the image for the adipose tissue because of the characteristics of the device used, made it impossible to measure subcutaneous, intramuscular and uropygium region fat from the 21st to the 49th day of age. Therefore, it was impossible to view the predetermined regions in the quail body at any of the evaluated ages. Possibly due to the form of the adipose tissue deposition in the quails and the frequency of the transducer (7.5 MHz) used interfered in the evaluation not be suitable for the species. Theoretically, it is necessary to increase the transducer frequency to enable the measurement of this variable.

The increase in the energy of the diets promoted a reduction in mineral matter deposition up to 21 days of age and the level of body protein deposited up to 35 days of age. It is possible that the lower consumption of nutrients has influenced the deposition of calcium in quails since, in the initial phase, the demand for this mineral is high, influencing bone deposition and the mineral matter. According to Shahryari et al.^16^, high levels of fat in diets reduces calcium retention, bone ash content and bone calcium, interfering with mineral metabolism. According to the authors, it formed insoluble soaps during the digestion of nutrients, influencing the metabolism of fatty acids and adversely affecting the rate of calcium absorption.

At 21 days of age, the relative weight of the liver was reduced (*P* < 0.01) at levels above 2900 kcal ME/kg ratio, while it increased the weight of the gizzard with increasing levels of metabolizable energy (*P* < 0.05). Since the lower ME concentration in the diet increased food retention time, improved nutrient intake and improved their metabolism by the organ, causing an increase in the size of the organs. These results corroborate those found by Hussein et al.^17^, where the authors also observed an increase in liver weight in diets with lower energy content. While Önel et al.^18^, observed that the increase in the energy content of the diet increased the time the feed remained in the intestine, reflecting in heavier organs.

At 35 days, there was a reduction in the gizzards size, pancreas, and proventriculus as the energy levels in the diet increased; carcass fat levels rose. Possibly, the reduction in FI promoted a reduction in these organs because of the smaller volume of feed to be digested and metabolized in birds that consumed higher energy density. The greater deposition of protein (63.84 *vs.* 53.22%) also reinforced this at 35 days and mineral matter (9.62 *vs*. 8.87%) at 21 days in the body of the quails that consumed lower levels of ME.

These results are reinforced by the information in Oliveira et al.^19^, who observed that the weight of the pancreas could present changes in the digestive capacity of the bird because of the high correlation with the activity of pancreatic digestive enzymes. Thus, birds that consumed diets with lower energy density, 2700 kcal ME/kg in phase I and 2900 kcal ME/kg in phase *II*, increased FI. However, they had a higher speed of passage of the chyme in the digestive tract, being compensated by the greater production of digestive enzymes, allowing greater deposition of nutrients as protein and maintaining the weight gain in these birds.

The higher energy levels also provided an increase in the adipose tissue deposition because of the excessive supply of energy for the quails, justifying the greater fat deposition after 35 days of age. According to Muniz et al.^13^, the rate of abdominal fat is directly influenced by the age of birds and is highly correlated with dietary fat; thus, excess dietary fat may reflect the total abdominal and body fat of the bird.

At 49 days of age, the development of the organs stabilized, affecting the physiological maturation of the quails. Fat deposition increased in the diet with more energy, a fact already expected at this stage. Jahanian and Edriss^20^ evaluated different levels of protein and energy on the carcass yield of meat quail from eight to 49 days of age, and they observed significant results on the weight of abdominal fat and other analyzed variables. Thus, there is evidence of increased ME (3200 kcal ME/kg) about FI and adipose tissue deposition in quail.

As BW were similar between treatments, similar carcass and cut yields were expected. Greater wing development may indicate greater fat deposition or greater of these parts (wing) development in the quail, a fact to be better investigated in future works.

Regarding the sensory analysis, ME levels did not influence the meat attributes, possibly because of the low incidence of intramuscular fat that is characteristic of the *M. pectoralis major*. In birds, there is a greater accumulation of fat in the abdominal, visceral and rump regions; as a result, there is a lower content of marbling fat in the assessed muscle^21^. The *M. pectoralis major* it is predominantly formed by white glycolytic fibers that present greater growth in their diameter, reflecting a lower content of marbling fat^22^, a fact that further justifies the similarities between the treatments.

As the quail were slaughtered within the recommended slaughter time, between 42 and 49 days of age, there was no time for the intramuscular fat to be deposited. Abou-Kassem et al.^23^ verified the increase in intramuscular fat with increasing age at slaughter of quail.

As reported by Silva^8^, the fat deposition in the body presents temporal variation, being deposited primarily the internal fat (abdominal, pelvic and coelomic cavity); subsequently, intermuscular fat; subcutaneous fat and finally, intramuscular fat (marbling). Linked to this, the glycolytic fibers (white muscle) predominant in birds, which are, which have a lower lipid content, when compared to oxidative fibers (red muscle), characteristic in cattle, which have a positive correlation between the number of red fibers and the muscle marbling rate, showing greater marbling in oxidative muscles^24^.

Increasing the energy level of the feed from 2700 to 3100 kcal of ME/kg in phase *I* and from 2900 to 3200 kcal of ME/kg in phase *II*, reduces FI and improves quail FE. However, it increases the level of fat in the carcass after 35 days of age, without harming the development of the birds, as well as the physical and sensory characteristics of the meat.

The ultrasound technique in vivo using the area M. *pectoralis major* in quails by the transversal-sectional reading, considering the age and energy level of the diet, it made it possible to predict the muscle area with greater reliability.

## Methods

### Ethics statement

This experiment was approved by the Ethics Committee on Animal Use (CEUA number 08/2017) of the Federal University of Sergipe, Brazil. All animal procedures were performed according to the approved protocol and are in accordance by ARRIVE guidelines^25^.

### Experimental design

Two hundred and seventy of dual-purpose domestic quail (*Coturnix japonica*), males and females (mixed sex), were used. The birds were floor-reared until they were six days old^6^. During this period were also attended the nutritional requirements and the environmental parameters of the species as a function of age according to the Silva and Costa^26^.

At seven days of age, the quail of both sexes were weighed and transferred to metabolic cages (0.60 × 0.50 × 0.45 m) equipped with gutter-type feeders and drinking troughs nipples. The birds were kept in cages from 7 to 49 days of age, totaling 42 days of the experiment.

A completely randomized design was adopted, comprising three treatments, five replicates and 18 birds/cage, with the cage being considered the experimental unit. The treatments comprised three increasing levels of metabolizable energy (ME): Phase *I*—7 to 21 days of age, containing energy levels of 2700; 2900 and 3100 kcal of ME/kg of feed and phase *II*—21 to 49 days, containing diets of 2900; 3050 and 3200 kcal ME/kg.

The experimental diets (Table 7) were formulated to meet the nutritional requirements of quail in phase *I*, from 7 to 21 days of age; and in phase *II*, from 21 to 49 days; based on the recommendations in the Silva and Costa^26^; Rostagno et al.^27^. The diets were isoproteics, all diets maintained the same protein pattern and the quail received feed and water ad libitum throughout the experimental period.

**Table 7:** Composition of experimental diets.

Ingredients (kg)	Metabolizable energy levels (kcal/kg)					
	Phase I			Phase II		
	2700	2900	3100	2900	3050	3200
Corn	45.957	41.296	36.637	60.720	57.645	54.570
Soybean meal	45.780	46.590	47.400	35.600	35.840	36.080
Wheat bran	4.000	4.000	4.000	–	–	–
Soybean oil	0.188	4.041	7.894	0.450	3.265	6.081
Dicalcium phosphate	1.005	1.012	1.018	0.897	0.904	0.912
Calcite limestone	1.803	1.798	1.793	1.503	1.503	1.502
Sodium chloride	0.392	0.393	0.393	0.345	0.345	0.346
Premix vit. and min.^1^	0.200	0.200	0.200	0.200	0.200	0.200
l-Lysine HCl	0.103	0.092	0.080	–	–	–
DL-Metionine	0.378	0.383	0.387	0.225	0.231	0.238
l-Threonine	0.195	0.197	0.198	0.060	0.066	0.071
Total	100.00	100.00	100.00	100.00	100.00	100.00

Chemical composition						
ME (kcal/kg)	2700	2900	3100	2900	3050	3200
Crude protein (%)	25.00	25.00	25.00	20.93	20.80	20.67
Dig. Lysine (%)	1.370	1.370	1.370	1.020	1.020	1.020
Dig. Met. + Cist. (%)	1.040	1.040	1.040	0.800	0.800	0.800
Dig. Tryptophan (%)	0.295	0.297	0.299	0.239	0.239	0.238
Dig. Threonine (%)	1.040	1.040	1.040	0.780	0.780	0.780
Available phosphorus (%)	0.320	0.320	0.320	0.270	0.270	0.270
Calcium (%)	0.850	0.850	0.850	0.700	0.700	0.700
Sodium (%)	0.170	0.170	0.170	0.150	0.150	0.150

^1^Warranty levels per kg of product: Folic acid (min.) 200 mg; pantothenic acid (min.) 5350 mg; copper (min.) 4000 mg; iron (min) 20 g; iodine (min.) 1500 mg; manganese (min.) 75 g; niacin (min.) 19.9 g; selenium (min.) 250 mg; Vit. A (min.) 8,000,000 IU; Vit. B12 (min.) 10,000 mcg; Vit. B2 (min.) 4000 mg; Vit. B6 (min.) 1000 mg; Vit. D3 (min) 2,000,000 IU; Vit. E (min.) 15,000 IU; Vit. K3 (min.) 2000 mg; zinc (min.) 50 g. Dig. Met. + Cist: digestible Methione + Cystine.

The metabolizable energy level was calculated considering the values of the digestibility coefficient of nutrients for birds contained in Rostagno et al.^27^.

For performance analysis, quails were weighed at seven, 21, 35 and 49 days to determine body weight—BW (g), average weight gain—AWG (g/period), feed intake—FI (g) and feed efficiency—FE (ratio of weight gain and feed intake). The mortality record was performed daily for correction.

To monitor the thermal environment a thermohygrometer (HTR-157 RS 232/Datalogger) was used, and the thermometer was placed in the center of the installation at median height of the cages. The mean minimum and maximum temperatures got were 24.8 ± 0.60 °C and 30.3 ± 0.71 °C, respectively, and the relative humidity was 45 ± 1.48%.

### Ultrasound technique

**Figure 1:**
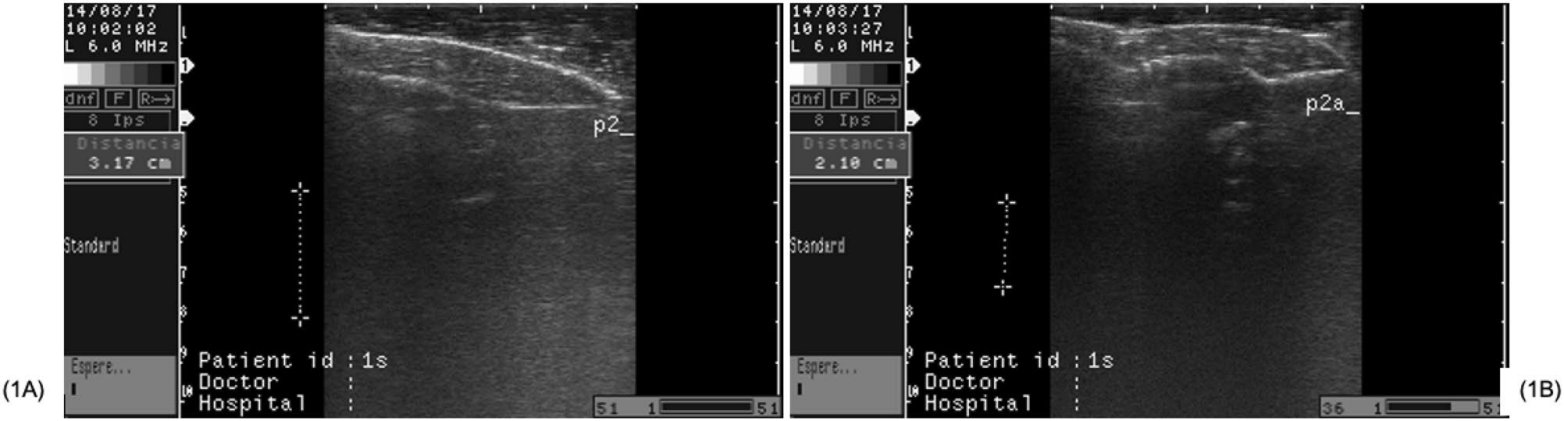
The ultrasound images of M. *pectoralis major*, in domestic quail (*Coturnix japonica*). (**A**) Longitudinal positions; (**B**) Transverse positions.

At 21, 35 and 49 days old, three quails from each experimental unit were selected, based on the AWG of the treatment, for in vivo ultrasonography analysis (n = 15 quails from each treatment). Images of the M. *pectoralis major* were using the Echo Honda ultrasound equipment (model HS-1500 VET, Honda Electronics Co, Japan) with a 50 mm wide multi-frequency linear transducer with a frequency of 7.5 MHz. The linear transducer was positioned in the ventral portion of the quail breast for reading in the longitudinal position (Fig. 1A) and another in the transverse position (Fig. 1B).

**Figure 2:**
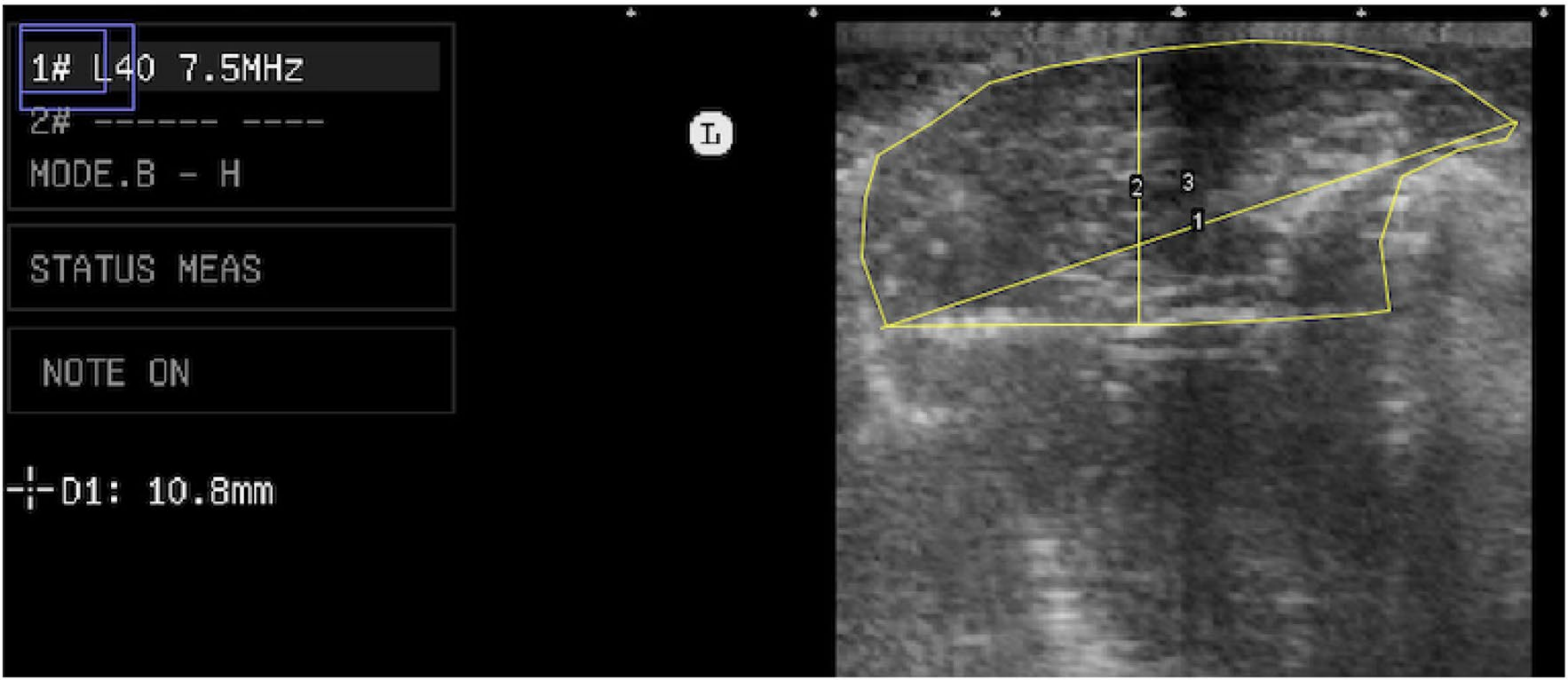
Image analysis using the ImageJ software the area (cm^2^), length (cm) and depth (cm) of the M. *pectoralis major* in the transverse positions in domestic quail (*Coturnix japonica*). 1—pectoral muscle length (cm); 2—pectoral muscle depth (cm); 3—pectoral muscle area (cm^2^)—the entire area bounded by the outer line.

From the ultrasound images, the area (cm^2^), width (cm) and depth (cm) of the M. *pectoralis major* in the longitudinal and transverse positions (Fig. 2). Based on this information, were obtained prediction equations to determine the area, width and depth of the M. *pectoralis major* as a function of quail age and metabolizable energy levels in the diets.

### Relative weight of viscera and body chemical composition

After ultrasound, the quails (n = 15 birds from each treatment) were submitted to a six-hour fasted and were euthanized by cervical dislocation^28^. The quails were plucked, opened by the back, eviscerated, followed by carcass hygiene and weighed. Then, the heart, liver, pancreas, proventriculus, gizzard, intestine (both small and large intestine) and abdominal fat were removed and weighed to calculate their relative weight^29^. Carcasses and organs were frozen and stored for further analysis of body composition.

For body chemical composition, the whole carcasses with feathers and organs were placed in an autoclave, then ground, weighed, homogenized and taken to a forced ventilation oven at 55 °C for 72 h. The proximal chemical composition was determined according to the AOAC method^30^ for dry matter (Method 934.01), mineral matter (Method 942.05), organic matter (Method. 2.7.08), crude protein (Method 990.03) and ether extract (Method 920.39).

### Quantitative and qualitative parameters of the carcass

At 49 days of age, four birds from each experimental unit (n = 20, being two females and two males from replicate) were slaughtered. After slaughter, the birds were plucked, opened by the back, eviscerated, and the carcasses were sanitized. A carcass was considered being the whole bird with skin, eviscerated, being the head, neck, shin and feet were removed; later, the breast (*M. pectoralis major*), thigh + drumstick and wing cuts were removed to analyze their yield of these cuts. The carcass was analyzed both by its absolute weight and by its yield^31^.

The pH in *post-mortem* was measured directly in the M. *pectoralis major*, about five minutes and 24 h after slaughter using a portable pH-meter with penetration electrode (Hanna® model HI 99,163, Hanna Instruments, USA), as suggested by Gontijo et al.^32^.

### Sensory analysis

For the sensory analysis, fifteen birds were slaughtered per treatment (n = 45), respecting all current norms for the procedure.

To prepare the samples used in the sensory analysis, the carcasses were cleaned and frozen at a temperature 5 °C for 24 h. Afterwards, they were thawed and marinated in 10% brine for 10 min. The breast fillet was then placed in aluminum foil packaging for cooking on an electric grill until reaching a temperature of 72 °C, measured using a digital skewer thermometer. After cooking, the breast was cut into individual samples of 1.5 cm, then wrapped in a container with a coded. Then, they were stored on a heated plate (60 °C) until the beginning of sensory tests.

Eighty-three untrained consumers were selected since these consumers represent the general population, the target audience of the study. At the time of evaluation, each taster remained in a booth, isolated from the other tasters. The tasters received an eight-point hedonic scale form: where 1 represents: I disliked a lot, and 8 represents: I liked a lot, as described by Dutcosky^33^. Then, the tasters received a container containing a coded sample of each treatment. Water and cream cracker were offered to neutralize the palate between tasting.

### Statistical analysis

The performance data, organ yield, body chemical composition and quantitative and qualitative parameters of the carcass were submitted to the Kruskal Wallis test at a 5% probability level to verify normality. Data with normal distribution were subjected to ANOVA with the aid of the Statistical Analysis System^34^, and when significant, the Tukey Test was used to observe differences between treatments. A 5% probability was considered for all variables.

For the analysis of the data from the ultrasound of the birds, the SAS^14^ program was used. The measurements were analyzed using multiple linear regression (Proc. REG.) associating age and energy level of the ration: Y_ij_ = β_0_ + β_1_X_i1_ + β_2_X_i2_ + €_ij_; considering Y: the evaluated measure; β_0_: intercept of the plane with the axis; β_1_ and β_2_: regression coefficients; X_i1_: considered age; X_i2_: energy level; €_ij_: independent random errors.

In the sensory analysis, the data were submitted to ANOVA with the aid of the statistical program PAST (version 2.04)^35^and evaluated using the Tukey test at the 5% probability level.

## Data Availability

The data that support this study cannot be publicly shared due to ethical or privacy reasons and may be shared upon reasonable request to the corresponding author if appropriate.
